# Determination of *in vitro* immunotoxic potencies of a series of perfluoralkylsubstances (PFASs) in human Namalwa B lymphocyte and human Jurkat T lymphocyte cells

**DOI:** 10.3389/ftox.2024.1347965

**Published:** 2024-03-14

**Authors:** Aafke W. F. Janssen, Wendy Jansen Holleboom, Deborah Rijkers, Jochem Louisse, Sjoerdtje A. Hoekstra, Sanne Schild, Misha F. Vrolijk, Ron L. A. P. Hoogenboom, Karsten Beekmann

**Affiliations:** ^1^ Wageningen Food Safety Research (WFSR), Wageningen University and Research, Wageningen, Netherlands; ^2^ European Food Safety Authority, Parma, Italy; ^3^ Department of Pharmacology and Toxicology, Maastricht University, Maastricht, Netherlands

**Keywords:** PFASs, immunotoxicity, Namalwa cells, Jurkat cells, relative potency factors, *in vitro* methods

## Abstract

Exposure to PFASs is associated to several adverse health effects, such as immunotoxicity. Immunotoxic effects of PFOA and PFOS, including a reduced antibody response in both experimental animals and humans, have been reported. However, there is limited understanding of the underlying mechanisms involved. Moreover, there is only a restricted amount of immunotoxicity data available for a limited number of PFASs. In the current study the effects of 15 PFASs, including short- and long-chain perfluorinated carboxylic and sulfonic acids, fluorotelomer alcohols, and perfluoralkyl ether carboxylic acids were studied on the expression of recombinant activating gene 1 (*RAG1*) and *RAG2* in the Namalwa human B lymphoma cell line, and on the human IL-2 promotor activity in Jurkat T-cells. Concentration-response data were subsequently used to derive *in vitro* relative potencies through benchmark dose analysis. *In vitro* relative potency factors (RPFs) were obtained for 6 and 9 PFASs based on their effect on *RAG1* and *RAG2* gene expression in Namalwa B-cells, respectively, and for 10 PFASs based on their inhibitory effect on IL-2 promotor activity in Jurkat T-cells. The most potent substances were HFPO-TA for the reduction of *RAG1* and *RAG2* gene expression in Namalwa cells (RPFs of 2.1 and 2.3 respectively), and PFDA on IL-2 promoter activity (RPF of 9.1). RAG1 and RAG2 play a crucial role in V (D)J gene recombination, a process for acquiring a varied array of antibodies crucial for antigen recognition. Hence, the effects observed in Namalwa cells might indicate a PFAS-induced impairment of generating a diverse range of B-cells essential for antigen recognition. The observed outcomes in the Jurkat T-cells suggest a possible PFAS-induced reduction of T-cell activation, which may contribute to a decline in the T-cell dependent antibody response. Altogether, the present study provides potential mechanistic insights into the reported PFAS-induced decreased antibody response. Additionally, the presented *in vitro* models may represent useful tools for assessing the immunotoxic potential of PFASs and prioritization for further risk assessment.

## Introduction

Poly- and perfluoralkyl substances (PFASs) are manmade chemicals of which many are highly persistent in the environment due to their resistance to degradation. Humans are exposed to PFASs via the environment and food, and several PFASs have been shown to accumulate in the body due to their limited clearance, raising concerns for human health (Pérez et al., 2013; Poothong et al., 2020; ATSDR, 2021). Animal and human epidemiological studies indeed demonstrated that exposure to PFASs is associated with a wide range of adverse effects, including hepatoxicity, developmental toxicity, a decrease in thyroid hormone levels, and immunotoxicity (EFSA CONTAM Panel and Scientific Opinion, 2018; EFSA CONTAM Panel and Scientific Opinion, 2020; ATSDR, 2021). In its most recent opinion on PFASs, the European Food Safety Authority (EFSA) CONTAM panel reassessed the health risks posed by PFASs and considered the immunotoxic effects as the most critical human health effect (EFSA CONTAM Panel and Scientific Opinion, 2020). Based on human data showing a negative association between plasma levels of the sum of perfluorooctanoic acid (PFOA), perfluorononaoic acid (PFNA), perfluorohexane sulfonate (PFHxS) and perfluorooctane sulfornate (PFOS), and antibody titres against diphteria in one-year-old infants, EFSA defined a health-based guidance value (tolerable weekly intake (TWI)) of 4.4 ng/kg bw for the four PFASs (Abraham et al., 2020; EFSA CONTAM Panel and Scientific Opinion, 2020). Since there were insufficient data to determine relative potency factors (RPFs) for the effects of the individual PFASs on the immune system, equal potencies were assumed for all four PFASs (EFSA CONTAM Panel and Scientific Opinion, 2020).

Currently, the majority of the immunotoxicity data is only available for a limited number of PFASs, primarily the legacy PFASs, such as PFOA and PFOS. Considering the concerns of adverse effects to both health and the environment, PFOS was included in the Stockholm Convention in 2009, followed by PFOA in 2019 and by PFHxS, its salts and PFHxS-related compounds in 2022, to restrict their production and use (European Parliament, Council of the European Union, 2019; European Commission, Directorate-General for Environment, 2020; UNEP, 2020; UNEP, 2022). As long-chain PFASs generally appear more bio-accumulative than short-chain PFASs, long-chain perfluorinated carboxylic acids (C9-C21) are currently also considered for inclusion in the Stockholm convention. Due to the elimination of PFOA and PFOS, fluorotelomer industries reformulated and shifted towards the production of polyfluorinated compounds with shorter alkyl chain lengths (e.g., perfluorobutane sulfonate (PFBS), perfluorobutanoic acid (PFBA), fluorotelomer alcohols (FTOH) and perfluoralkyl ether carboxylic acids, e.g., hexafluoropropylene oxide dimer acid (HFPO-DA)) (UNEP, 2013; Lu et al., 2019). Although these short- and long-chain PFASs have similar physical and chemical properties due to their unique C-F bonds, short-chain PFASs are more mobile and persistent in aquatic ecosystems (Brendel et al., 2018). To date, knowledge regarding the immunotoxic effects of these PFAS substitutes is limited, but recent animal studies suggest that some of these compounds also may affect the immune system (Wang et al., 2019; U.S. Environmental Protection Agency, 2021; Rushing et al., 2017; Weatherly et al., 2021).

**FIGURE 1:**
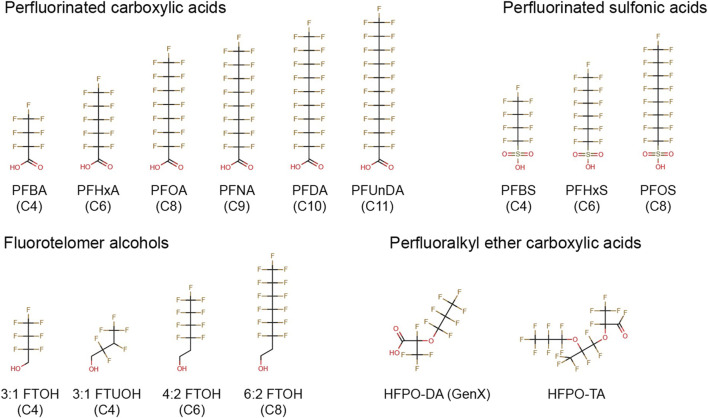
Chemical structures of PFASs tested in the present study. PFBA, perfluorobutanoic acid; PFHxA, perfluorohexanoic acid; PFOA, perfluorooctanoic acid; PFNA, perfluorononanoic acid; PFDA, perfluorodecanoic acid; PFUnDA, perfluoroundecanoic acid; PFBS, perfluorobutane sulfonate; PFHxS, perfluorohexane sulfonate; PFOS, perfluorooctane sulfonate; 3:1 FTOH, 2,2,3,3,4,4,4-heptafluoro-1-butanol; 3:1 FTUOH, 2,2,3,4,4,4-hexafluoro-1-butanol; 4:2 FTOH, 3,3,4,4,5,5,6,6,6-nonafluoro-1-hexanol; 6:2 FTOH, 3,3,4,4,5,5,6,6,7,7,8,8,8-tridecafluoro-1- octanol; HFPO-DA, hexafluoropropylene oxide dimer acid; HFPO-TA, hexafluoropropylene oxide trimer acid.

In the current study we studied the effects of a diverse group of 15 PFASs, including short- and long-chain perfluorinated carboxylic and sulfonic acids, fluorotelomer alcohols, and perfluoralkyl ether carboxylic acids (Figure 1), in two immune cell models. As the reduction in antibody levels upon PFAS exposure have been shown to be mediated by decreases in T-cell dependent antibody responses (TDAR) (Yang et al., 2002; Loveless et al., 2008; DeWitt et al., 2008; Dewitt et al., 2009; De Guise and Levin, 2021; Peden-Adams et al., 2008; Zheng et al., 2009; Dong et al., 2009; Dewitt et al., 2016) as well as T-cell independent antibody responses (TIAR) (Peden-Adams et al., 2008; Vetvicka and Vetvickova, 2013; Dewitt et al., 2016), we assessed the possible direct effects of selected PFASs on B- and T-cells involved in these processes. In our previous study, it was demonstrated that exposure of human B lymphoma Namalwa cells to PFOA, PFOS, PFNA or PFHxS reduced the expression of recombination-activating genes (RAG1 and RAG2) (Janssen et al., 2022). These effects may indicate a possible PFAS-induced impairment of the development of a diverse set of B-cells for adequate antigen recognition, as RAG1 and RAG2 play a role in the recombination of the so-called V (D)J genes, a process that is required to obtain the diverse set of antibodies for effective antigen recognition.

Besides B-cells, also T-cells are potential direct targets of PFASs, which may result in a decreased TDAR. Recently, the IL-2 Luc assay has been validated (Kimura et al., 2020a) and accepted by the OECD (OECD/OCDE, 2023) to assess immunotoxic effects of chemicals on T-cell activation. In the current study, a related IL-2 reporter gene assay has been used to assess effects of PFASs on T-cell activation. IL-2 is a cytokine that is primarily produced by T-cells following their activation by an antigen and plays an important role in the TDAR. During T-cell dependent B-cell activation, activated T-cells secrete IL-2, thereby promoting B-cell proliferation, their differentiation towards plasma cells and immunoglobulin production (Mingari et al., 1984; Le Gallou et al., 2012; Hipp et al., 2017). A chemical-induced TDAR decrease can be caused by a chemical-induced inhibition of T-cell activation, for instance, through the suppression of calcineurin activity as in the adverse outcome pathway no. 154 (http://aopwiki.org/aops/154).

In the current study, the effect of 15 PFASs on *RAG1* and *RAG2* gene expression in Namalwa B-cells, and on IL-2 promotor activity in Jurkat T-cells was assessed in order to provide more mechanistic insight into the mode of action of the antibody lowering effects of PFASs. In addition, concentration-response data on the reduction in *RAG1* and *RAG2* gene expression, and IL-2 promotor activity were analyzed using PROAST software to assess possible *in vitro* potency factors for the 15 PFASs.

## Materials and methods

### Chemicals

The following PFASs were tested in the current study: perfluorobutanoic acid (PFBA), perfluorohexanoic acid (PFHxA), perfluorooctanoic acid (PFOA), perfluorononaoic acid (PFNA), perfluorodecanoic acid (PFDA), perfluoroundecanoid acid (PFUnDA), perfluorobutane sulfonate (PFBS), perfluorohexane sulfonate (PFHxS), perfluorooctane sulfornate (PFOS), 2,2,3,3,4,4,4-heptafluoro-1-butanol (3:1 FTOH), 2,2,3,4,4,4-hexafluoro-1-butanol (3:1 FTUOH), 3,3,4,4,5,5,6,6,6-nonafluoro-1-hexanol (4:2 FTOH), 3,3,4,4,5,5,6,6,7,7,8,8,8-tridecafluoro-1-octanol (6:2 FTOH), hexafluoropropylene oxide dimer acid (HFPO-DA, also known as GenX), and hexafluoropropylene oxide trimer acid (HFPO-TA) (Figure 1). In addition, the FOXO1 inhibitor AS1842856, the mycotoxin deoxynivalenol (DON), and the calcineurin inhibitor FK506 (Tacrolimus) were used as model compounds. All stocks were prepared in 100% dimethyl sulfoxide (DMSO HybriMax, Sigma-Aldrich), except for DON which was dissolved in ethanol. Additional details regarding suppliers, purity, catalog numbers and the highest maximum concentrations tested in the present study is presented in Supplementary Datasheet 1.

### Cell lines and cell culture

The human Burkitt’s lymphoma cell line Namalwa was purchased from Sigma-Aldrich (Zwijndrecht, Netherlands). Cells were cultured in a humidified incubator (37°C, 5% CO_2_) in RPMI1640 (Gibco, Thermo Fisher Scientific, Waltham, MA) supplemented with 10% heat-inactivated fetal bovine serum (FBS; Gibco), 1% sodium pyruvate (Sigma–Aldrich), 1% nonessential amino acids (NEAA; Gibco) and 1% penicillin–streptomycin (Sigma–Aldrich). After the Namalwa cells reached a consistent growth rate (approximately after 7–10 days), the quantity of heat-inactivated FBS was decreased to 2% to reduce PFASs binding to proteins and thereby increase the amount of substances available for cell exposure. Namalwa cells were sub-cultured twice a week, each time diluted to 0.5 × 10^6^ viable cells/mL. For experiments, Namalwa cells with a passage number between 10 and 22 were used.

The human IL-2 luciferase reporter Jurkat cell line was purchased from BPS Bioscience, Inc., (San Diego, CA). Cells were cultured in a humidified incubator (37°C, 5% CO_2_) in RPMI1640 (Gibco) supplemented with 10% heat-inactivated FBS (Gibco), 1% sodium pyruvate (Sigma–Aldrich), 1% NEAA (Gibco) and 1% penicillin–streptomycin (Sigma–Aldrich). Upon the first passage, 1 mg/mL Geneticin (Thermo Fisher Scientific) was added to the culture medium. For exposure studies, FBS was eliminated from the medium according to manufacturer instructions. The IL-2 luciferase reporter Jurkat cells were sub-cultured two to three times per week, each time diluted to 0.3 × 10^6^ viable cells/mL. For experiments, IL-2 luciferase reporter Jurkat cells with a passage number between 7 and 19 were used.

### Cell viability studies

The effect of the PFASs, DON, CD3/CD28 Dynabeads (Thermo Fisher Scientific), and FK506 on cell viability was determined using the WST-1 assay. This colorimetric assay determines the cleavage of the tetrazolium salt WST-1 (4-[3-(4-iodophenyl)-2-(4-nitrophenyl)-2H-5-tetrazolio]-1,3-benzene disulfonate) to formazan by metabolically active cells. Namalwa cells were cultured in 96-wells plates at a density of 1 × 10^6^ cells/mL and subjected to increasing concentrations up to 100 µM PFASs or to increasing concentrations up to 10 µM DON for 48 h. IL-2 luciferase reporter Jurkat cells were cultured in 96-wells plates at a density of 2 × 10^5^ cells/mL and subjected to increasing concentrations up to 100 µM for PFASs or to increasing concentrations up to 0.1 µM FK506 for 24 h. Only for PFUnDA the highest tested concentration was 33 μM, because it was not soluble at higher concentrations. For the CD3/CD28 Dynabeads, beads-to-cells ratios of 1:1, 2:1, 4:1 and 8:1 were tested for 24 h. After exposure, WST-1 (Sigma-Aldrich) solution was added to the cell culture medium at a dilution of 1:10. After 1 h incubation in a humidified incubator (37°C, 5% CO_2_), the plate was shaken at 1,000 rpm for 1 min, and absorbance at 450 nm was measured (the background absorbance at 630 nm was subsequently subtracted during data analysis) using a Synergy HT Microplate Reader (BioTek, Winooski, VT). Two independent studies were conducted, each comprising three technical replicates per condition for PFASs exposure studies. Additionally, one independent study was performed for DON, FK506 and CD3/CD28 Dynabeads, with three technical replicates in each study. Cell viability is expressed as percentage of WST-1 transformation observed for the solvent control. For all treatment conditions, DMSO was used as a solvent control, except for DON where ethanol was used. The solvent concentration was 0.1% in all treatment conditions.

### Namalwa exposure for gene expression analysis

For gene expression studies, Namalwa cells were seeded in 24-well plates (Corning, NY) at a density of 0.5–1 × 10^6^ cells per well in 500 μL. Test chemicals were initially diluted from a 1000-fold concentrated stock to a twofold concentrated stock solution in assay medium. Subsequently, they were twofold diluted upon the addition to the Namalwa cell suspension, resulting in a final DMSO concentration of 0.1%. Each experiment included a solvent control (0.1% DMSO). Namalwa cells were subjected to a PFAS concentration range up to the highest non-cytotoxic concentration, causing less than 20% reduction in cell viability as determined by a reduction in mitochondrial activity using the WST-1 assay (100 μM for PFBA, PFHxA, PFOA, PFBS, PFHxS, PFOS, 3:1 FTOH, 3:1 FTUOH, 4:2 FTOH, 6:2 FTOH and HFPO-DA, 33 μM for PFNA, PFDA and HFPO-TA, and 10 μM for PFUnDA) for 48 h. The exposures of the Namalwa cells to PFASs were executed in four independent studies, each including exposure to the two positive controls 0.01 μM AS1842856, a FOXO1 inhibitor, and 3.3 μM DON for 48 h. After exposure, effects of the PFASs, AS1842856 and DON on expression of *GAPDH*, *RAG1,* and *RAG2* were assessed.

### RT-qPCR

After exposure of Namalwa cells to PFASs, AS1842856 or DON, the medium containing the cells were collected and centrifuged at 200 × g for 7 min to obtain a cell pellet. Subsequently, the Namalwa cell pellets were lysed using cell lysis buffer (RLT) and total RNA was extracted utilizing the RNeasy Mini Kit (Qiagen, Venlo, Netherlands). Next, 500 ng RNA was used to synthesize cDNA using the iScript cDNA synthesis kit (Bio-Rad Laboratories, Veenendaal, Netherlands). Gene expression alterations were assessed by RT-qPCR on a CFX384 real-time PCR detection system (Bio-Rad Laboratories) using SensiMix (Bioline; GC Biotech, Alphen aan den Rijn, Netherlands) as previously described (Janssen et al., 2022). Primer sequences were obtained from the Harvard PrimerBank and acquired from Eurogentec (Liège, Belgium), with the sequences provided in Table 1. Relative gene expression was determined using a standard curve generated from a serial dilution of pooled sample cDNA, and subsequently normalized to glyceraldehyde 3-phosphate dehydrogenase (GAPDH) gene expression. Gene expression changes following PFAS treatments were expressed as fold change relative to the gene expression measured for the solvent control. Concentration–response data were analyzed using PROAST software for benchmark dose (BMD) analysis, as described below.

**TABLE 1:** Primer sequences used for RT-qPCR.

Name	Primer sequence	
	Forward	Reverse
GAPDH	CTC​TGC​TCC​TCC​TGT​TCG​AC	TTA​AAA​GCA​GCC​CTG​GTG​AC
RAG1	TGC​ACA​GGA​AGT​TTA​GCA​GTG	ACG​GGC​AGT​GTT​GCA​GAT​G
RAG2	AGA​CTT​GGT​AGG​AGA​TGT​TCC​TG	TGT​ATG​AGC​GTC​CTC​CAA​AGA​G

### Jurkat exposure for IL-2 reporter activity

For IL-2 reporter activity studies, Jurkat IL-2 reporter T-cells were seeded in 96-wells plates (Corning, NY; 2 × 10^4^ cells per well in 100 μL). Test chemicals were initially diluted from a 1000-fold concentrated stock to a twofold concentrated stock solution in assay medium. Subsequently, they were twofold diluted upon the addition to the Jurkat IL-2 reporter T-cell suspension, resulting in a final DMSO concentration of 0.1%. Jurkat IL-2 reporter T-cells were first exposed to a PFAS concentration range up to the highest non-cytotoxic concentration, causing less than 20% reduction in cell viability as determined by a reduction in mitochondrial activity using the WST-1 assay (100 μM for PFBA, PFHxA, PFOA, PFBS, PFHxS, 3:1 FTOH, 3:1 FTUOH, 4:2 FTOH, 6:2 FTOH and HFPO-DA, 33 μM for PFNA, PFUnDA, PFOS and HFPO-TA, and 10 μM for PFDA) or a concentration range of FK506 (0.01, 0.1, and 1 μM) for 1 h prior to the addition of the CD3/CD28 Dynabeads (beads-to cells ratio of 8:1) for 24 h. Following incubation, the One-Step Luciferase Assay System (BPS Bioscience, San Diego, CA) was used to determine IL-2 reporter activity, according to manufacturer’s instructions. Luminescence was measured on a CLARIOstar plate reader (BMG Labtech, Ortenberg, Germany) and expressed as relative luciferase activity compared to the luminescense measured for Jurkat IL-2 reporter T-cells exposed to CD3/CD28 Dynabeads only.

### Benchmark dose (BMD) analysis using PROAST

Concentration-response modeling of the RT-qPCR data and IL-2 reporter activity data were performed using the BMD analysis software PROAST version 70.5 (National Institute for Public Health and the Environment 2018) in R (version 4.2.0), as previously described (Janssen et al., 2022). Average luciferase activity and gene expression values of triplicates with standard deviation used, can be found in Supplementary Datasheet 2 and 3, respectively. Of note, PROAST definitions are CES (critical effect size) and CED (critical effect dose), which are the same as BMR and BMC, respectively.

### Statistical analysis

Data are presented as mean ± SD. Comparisons between a single concentration of a compound (AS1842856, DON and CD3/CD28 Dynabeads) versus solvent control were analyzed using a two-tailed Student’s t test. A one-way ANOVA followed by Dunnett’s *post hoc* multiple comparison test was used for comparisons between Namalwa cells exposed to various concentrations of DON and solvent control, and Jurkat IL-2 reporter T-cells exposed to various dilutions of CD3/CD28 Dynabeads or FK506 and solvent control. *p* < 0.05 was considered as statistically significant. Prism software (version 9.3.1; Graphpad, San Diego, CA) was used for statistical analysis.

## Results

### Cell viability and gene expression studies in Namalwa B lymphocyte cells exposed to PFASs

**FIGURE 2:**
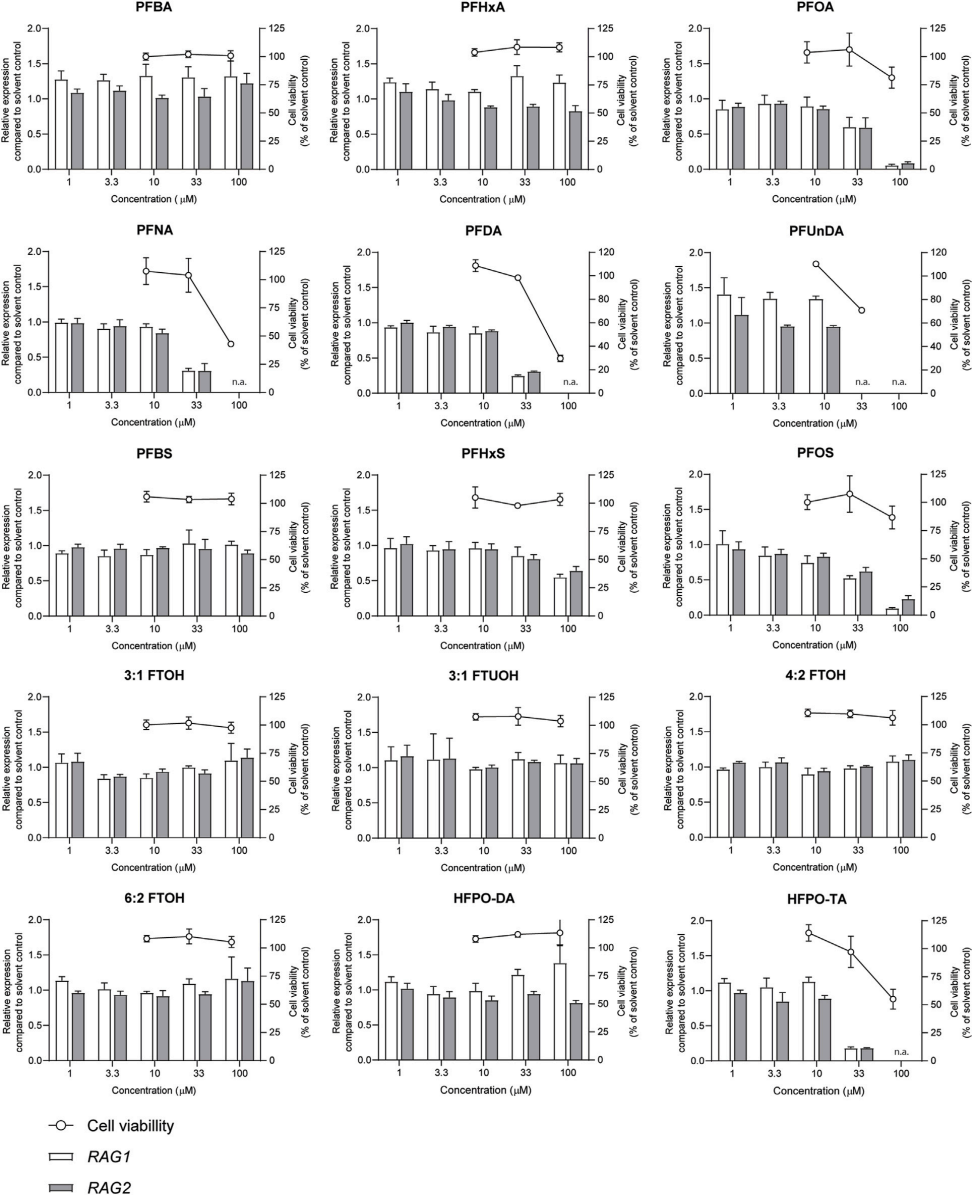
Concentration-dependent effects upon 48 h exposure to PFASs on Namalwa cell viability (circles, right Y-axes), and *RAG1* and *RAG2* gene expression (bars, left Y-axes). Gene expression levels of solvent control were set at one. The data points represent the mean ± SD of 2 independent studies, each performed in triplicate (*n* = 3) for the cell viability assay, and from one study with triplicate wells for the gene expression studies (*n* = 3). N.a.: concentrations that were excluded in gene expression studies and benchmark dose analysis.

The effects of a 48-h exposure of Namalwa cells to increasing concentrations of the PFASs (Figure 1) on cell viability was assessed using the WST-1 assay. Concentrations up to 100 µM could be tested for all PFASs, except for PFUnDA (up to 33 µM) due to its limited solubility in DMSO. Of the tested PFASs, PFUnDA was the most potently cytotoxic, i.e., causing a reduction in cell viability of 30% at a concentration of 33 µM. PFNA, PFDA, and HFPO-TA caused a decrease in cell viability at 100 µM. The remaining 11 PFASs did not cause a reduction of cell viability of 20% or more at the concentrations tested (Figure 2). In subsequent experiments, only concentrations of test substances were included that did not cause cytotoxicity in excess of at most 20% as determined by the WST-1 assay.

**FIGURE 3:**
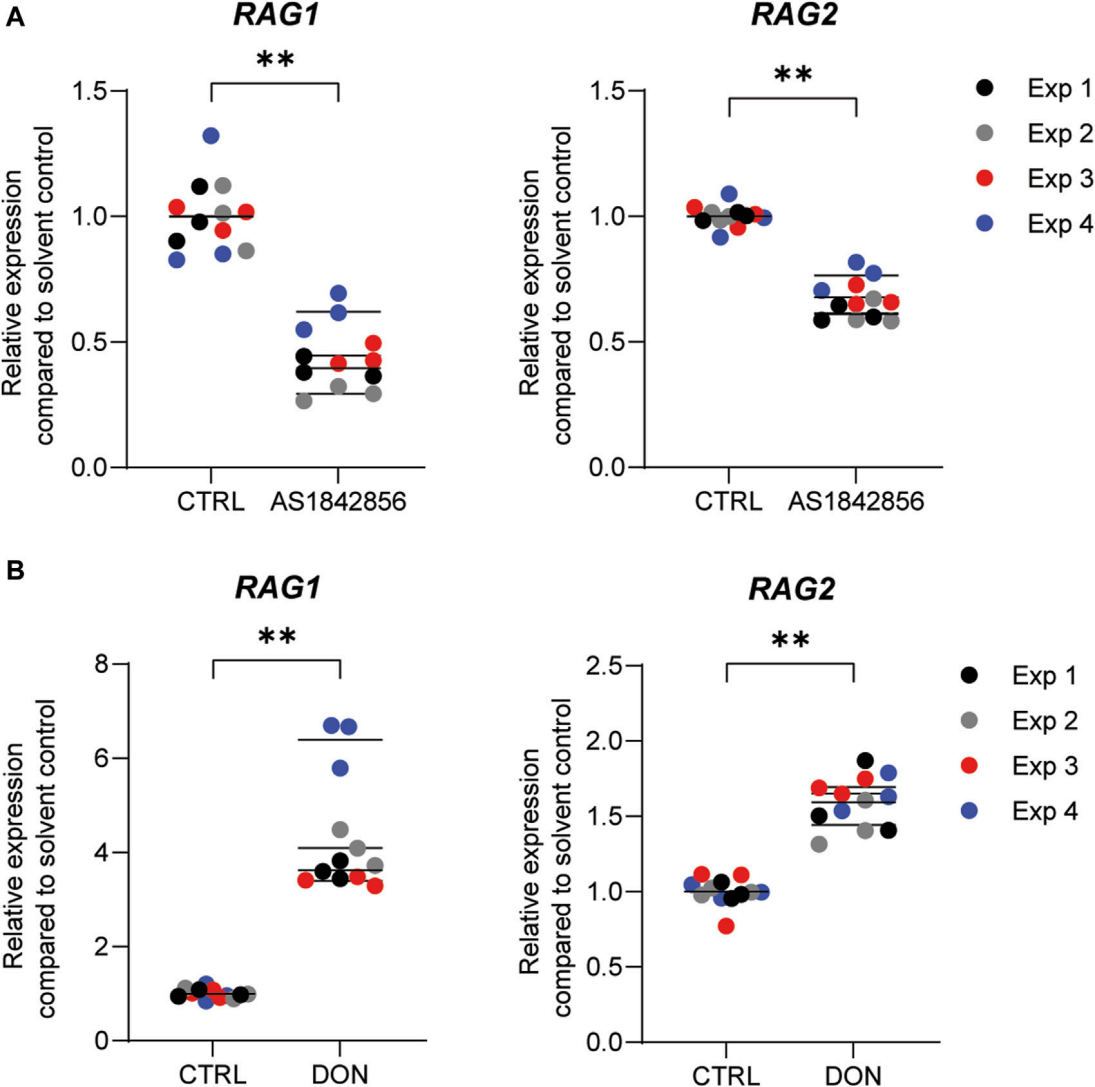
Effect of the model compounds AS1842856, a FOXO1 inhibitor, and Deoxynivalenol (DON), on *RAG1* and *RAG2* expression in Namalwa cells. Relative expression of *RAG1* and *RAG2* after incubation of Namalwa cells with **(A)** 0.01 µM AS1842856 or **(B)** 3.3 µM DON for 48 h. **p < 0.001. Gene expression levels of the solvent controls were set at one. Data are mean ± SD of 4 independent studies, each performed in triplicate (*n* = 12).

Previously, we demonstrated that PFOA, PFOS, PNFA and PFHxS caused a concentration-dependent decrease in the expression of *RAG1* and *RAG2* genes in Namalwa cells (Janssen et al., 2022). To determine whether *RAG1* and *RAG2* are also downregulated upon exposure to other PFASs, Namalwa cells were exposed to concentration ranges of the 15 PFASs for 48 h in four independent studies, each encompassing 2 to 4 PFASs. To ensure consistency across the four independent studies and enable comparison of *RAG1* and *RAG2* expression values between experiments, two model compounds were included in each independent experiment, i.e., one to increase expression and one to decrease expression. One of the model compounds is the FOXO1 inhibitor, AS1842856, which was previously shown to reduce *RAG1* and *RAG2* expression at a concentration of 0.01 µM (Janssen et al., 2022). The other model compound is DON, which was previously shown to increase phosphorylation of FOXO1 (Zhang et al., 2016). To assess whether this also translates into alterations in *RAG1* and *RAG2* gene expression, Namalwa cells were exposed to a concentration range of up to 10 µM of DON for 48 h. Only the concentration of 10 µM DON caused a reduction in cell viability, amounting to 50% (Supplementary Datasheet 1) and therefore 3.3 µM was selected as maximum concentration for further gene expression studies. Whereas 0.33 and 1 µM DON did not alter *RAG1* and *RAG2* gene expression, 3.3 µM DON caused an increase in *RAG1* and *RAG2* expression (Supplementary Datasheet 1). To that end, 0.01 µM AS1842856 and 3.3 µM DON were included in each independent experiment in which Namalwa cells were exposed to various concentration ranges of PFASs. As shown in Figure 3 similar responses on *RAG1* and *RAG2* gene expression in Namalwa cells exposed to AS1842856 and DON in the four independent experiments were observed, indicating that the effects of PFASs on *RAG1* and *RAG2* gene expression in Namalwa cells can be compared between independent experiments. Of the perfluorinated carboxylic acids, PFOA, PFNA and PFDA caused a clear reduction of both *RAG1* and *RAG2* expression. PFHxA only slightly decreased *RAG2* expression, though in a concentration-dependent manner. Of the tested perfluorinated sulfonic acids, PFHxS and PFOS caused a concentration-dependent decrease in the expression of *RAG1* and *RAG2* genes, whereas none of the fluorotelomer alcohols affected *RAG* expression. Contrary to HFPO-DA, HFPO-TA caused a reduction in *RAG1* and *RAG2* expression (Figure 2).

In order to gain insight into the relative potencies of PFASs in decreasing *RAG1* and *RAG2* expression in the employed assay system, gene expression data for the 15 PFASs in the Namalwa cells were used to perform concentration-response modeling using PROAST software, applying parallel curve fitting. A BMC, corresponding to a 10% decrease in *RAG1* and *RAG2* compared to background level, was assessed. Figure 6A illustrates the curves fitted by PROAST for the PFASs that yielded gene expression data for *RAG1* and *RAG2* that were adequate for BMD response modeling. Corresponding BMC_10_ values and RPFs are presented in Table 2. HPFO-TA was the most potent, being 2.1-fold and 2.3-fold more potent than PFOA in reducing *RAG1* and *RAG2* expression, respectively. PFNA and PFDA were also more potent than PFOA in reducing *RAG1* and *RAG2* gene expression (PFNA: 1.6-fold and 1.8-fold respectively; PFDA: 1.8-fold and 1.7-fold respectively). The potency of PFOS was slightly lower than that of PFOA, being 1.1-fold lower for *RAG1* and 1.4-fold lower for *RAG2.* Of the PFASs showing a reduction in *RAG* expression, PFHxS was the least potent PFAS in reducing *RAG1* expression, whereas the least potent PFASs causing a reduction in *RAG2* expression were PFHxA, HFPO-DA and PFBS (Table 2).

**TABLE 2:** Overview of BMC_10_ values (in µM) and related RPFs determined upon BMD modelling of data on reduction in RAG1 and RAG2 gene expression in Namalwa cells, and IL-2 promotor activity in Jurkat cells.

	Namalwa B-cells				Jurkat T-cells	
	*RAG1*		*RAG2*		IL-2 promotor activity	
PFAS	BMC_10_	RPF	BMC_10_	RPF	BMC_10_	RPF
PFBA	-	-	-	-	-	-
PFHxA	-	-	72.9 (50.2–160.0)	0.15 (0.1–0.2)	-	-
PFOA^a^	11.9 (9.6–14.7)	1^a^	10.8 (9.1–12.9)	1^a^	24.5 (19.0–31.1)	1^a^
PFNA	7.2 (6.1–8.6)	1.6 (1.5–1.8)	6.0 (5.2–6.9)	1.8 (1.7–1.9)	8.7 (6.8–11.0)	2.8 (2.4–3.3)
PFDA	6.5 (5.4–7.8)	1.8 (1.7–2.0)	6.3 (5.4–7.2)	1.7 (1.6–1.9)	2.7 (2.1–3.4)	9.1 (7.6–10.7)
PFUnDA	-	-	-	-	9.7 (7.4–12.6)	2.5 (2.1–3.0)
PFBS	-	-	123.2 (65.8–∞)	0.09 (0.0–0.2)	48.5 (35.7–72.1)	0.51 (0.3–0.7)
PFHxS	34.3 (28.7–41.1)	0.35 (0.3–0.4)	37.0 (31.6–43.6)	0.29 (0.2–0.3)	27.7 (21.6–35.1)	0.88 (0.7–1.0)
PFOS	13.7 (11.1–16.7)	0.87 (0.8–0.9)	15.4 (13.1–17.9)	0.70 (0.7–0.7)	5.1 (3.8–6.9)	4.8 (4.2–5.6)
3:1 FTOH	-	-	-	-	-	-
3:1 FTUOH	-	-	-	-	-	-
4:2 FTOH	-	-	-	-	-	-
6:2 FTOH	-	-	-	-	58.7 (41.4–104)	0.42 (0.2–0.6)
HFPO-DA	-	-	71.3 (49.5–150)	0.15 (0.1–0.2)	134 (64.0–∞)	0.18 (0.0–0.4)
HFPO-TA	5.8 (4.8–6.9)	2.1 (1.9–2.2)	4.8 (4.1–5.6)	2.3 (2.1–2.4)	4.8 (3.6–6.5)	5.1 (4.5–5.9)

^a^ PFOA, is used as an index chemical and potency was set at 1. 90% confidence intervals are presented between brackets.

### Cell viability and IL-2 promotor activity in Jurkat IL-2 reporter T-cells exposed to PFASs

**FIGURE 4:**
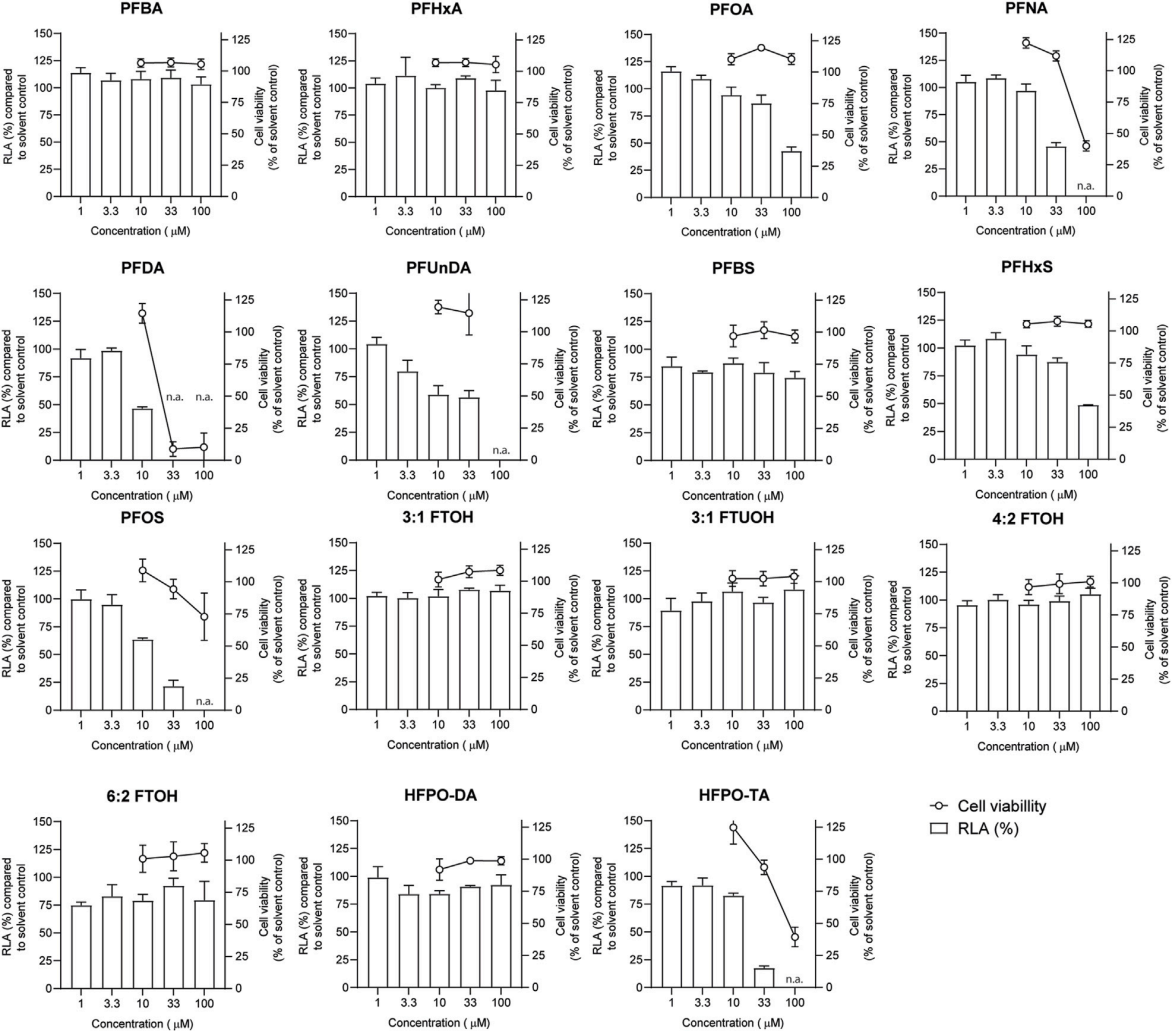
Concentration-dependent effects of 24 h exposure to PFASs on Jurkat IL-2 Luciferase Reporter cell viability (circles, right Y-axes), and relative luciferase activity ((RLA) bars, left Y-axes). For luciferase activity studies, Jurkat IL-2 reporter T-cells were exposed to a concentration range of PFASs for 1 h and subsequently CD3/CD28 Dynabeads at a 8:1 beads-to-cells ratio were added. The data points represent the mean ± SD of 2 independent studies, each performed in triplicate (*n* = 6) for the cell viability assays, and from triplicate wells for the luciferase activity studies (*n* = 3). N.a.: concentrations that were excluded in IL-2 promotor activity studies and benchmark dose analysis.

Jurkat T lymphocyte cells were exposed to increasing PFAS concentrations for 24 h and subsequently subjected to a WST-1 assay to assess the effect of exposure on cell viability (Figure 4). Concentrations up to 100 µM were tested for all PFASs, except for PFUnDA (up to 33 µM) due to its poor solubility in DMSO. Of the tested PFASs, PFDA was the most potently cytotoxic causing a decrease in cell viability of 90% at 33 μM, followed by PFNA, PFOS and HFPO-TA displaying cytotoxicity at 100 µM. The remaining 10 PFASs did not cause a reduction of cell viability of 20% or more at the concentrations tested, and in further experiments concentration ranges of test substances were used up to the highest concentration causing <20% reduction in cell viability.

**FIGURE 5:**
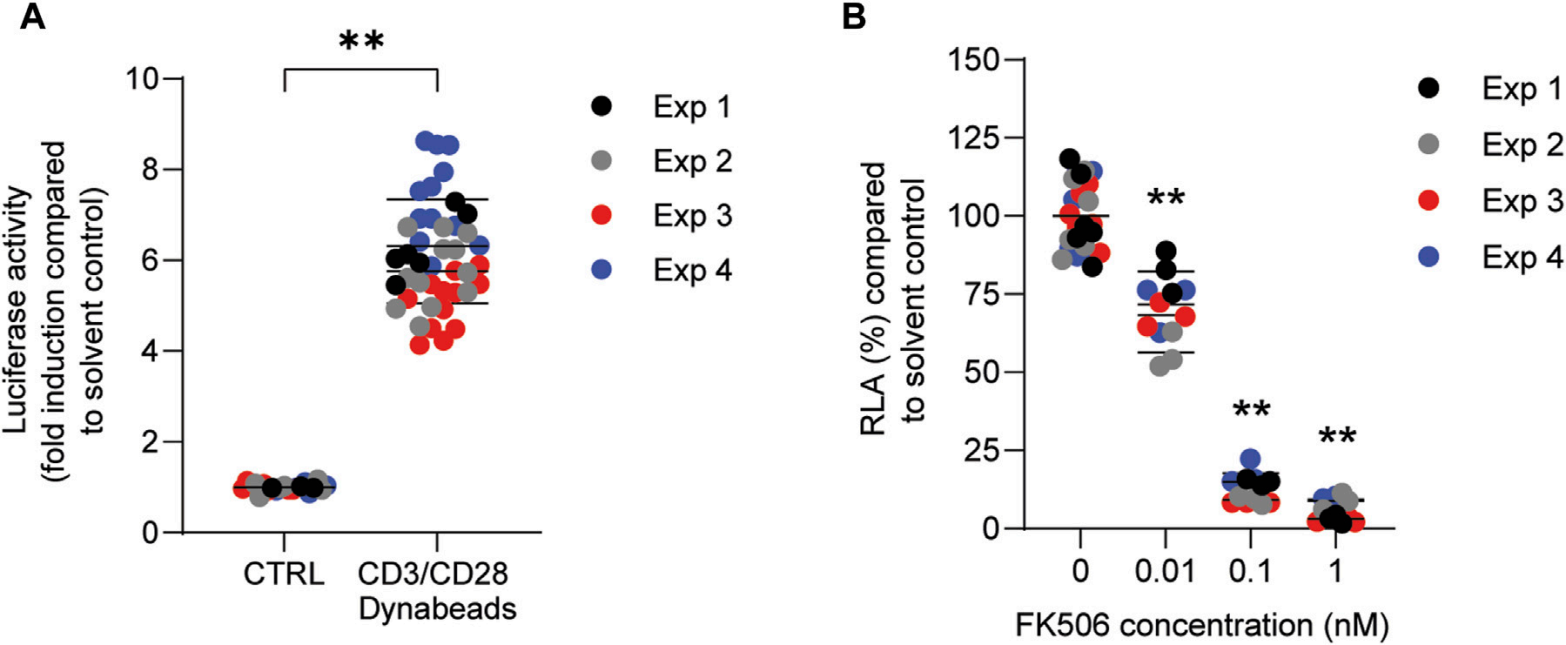
Effect of CD3/CD28 Dynabeads and FK506 on IL-2 luciferase activity in Jurkat IL-2 reporter T-cells. **(A)** Fold induction of IL-2 luciferase activity after exposure to 8:1 (beads-to-cells ratio) CD3/CD28 Dynabeads. **(B)** FK506 concentration-dependently decreases IL-2 luciferase activity. **p < 0.001. Data are mean ± SD of 4 independent studies, each performed at least in triplicate (*n* = ≥12).

In order to investigate whether PFASs can decrease IL-2 promotor activity, we first assessed which concentration of CD3/CD28 Dynabeads can induce the transcriptional activity of IL-2. Jurkat IL-2 reporter cells were incubated with 1:1, 2:1, 4:1, and 8:1 beads-to-cells ratio of CD3/CD28 Dynabeads for 24 h. None of these CD3/CD28 Dynabeads beads-to-cells ratios affected cell viability (Supplementary Datasheet 1) and were therefore used to assess effects on IL-2 promotor activity. A concentration-dependent increase in IL-2 promotor activity was observed upon stimulation with increasing beads-to-cells ratio of CD3/CD28 Dynabeads, with a 12-fold induction of IL-2 promotor activity at an 8:1 beads-to-cells ratio (Supplementary Datasheet 1). Therefore, for subsequent studies an 8:1 beads-to-cells ratio of CD3/CD28 Dynabeads was used to induce transcriptional activity of IL-2. To assess whether PFASs exposure affects IL-2 promotor activity, Jurkat IL-2 reporter T-cells were exposed to concentration ranges of the 15 PFASs for 1 h prior to the addition of CD3/CD28 Dynabeads, and the subsequent 24 h of exposure in four independent studies, each encompassing 2 to 4 PFASs. The induction of IL-2 luciferase activity by CD3/CD28 Dynabeads was similar in the four independent studies (Figure 5A). In addition to the induction of IL-2 promotor activity, also the model compound FK506 (Tacrolimus), a calcineurin inhibitor known to inhibit IL-2 luciferase activity (Kimura et al., 2018; Kimura et al., 2020b), was included in each independent experiment. Exposure of the IL-2 reporter T lymphocytes for 24 h to the non-cytotoxic (Supplementary Datasheet 1) concentrations 0.1, 1 and 10 nM FK506 decreased the relative luciferase activity, being inhibited with 64% at 0.1 nM FK506 and almost completely at 1 and 10 nM FK506 Supplementary Datasheet 1. In each of the independent experiments, the range of FK506 concentrations caused a similar reduction in IL-2 reporter activity, suggesting that response of the cells is comparable, and that the results of the different PFASs from the four independent experiments can be combined and compared (Figure 5B). Of the perfluorinated carboxylic acids, PFDA caused a reduction of IL-2 promotor activity at 10 µM and PFNA at 33 μM, and PFOA and PFUnDA decreased IL-2 promotor activity in a concentration-dependent manner over several concentrations. Of the tested perfluorinated sulfonic acids, PFHxS and PFOS caused a concentration-dependent reduction in IL-2 promotor activity, whereas none of the fluorotelomer alcohols affected IL-2 transcription. Of the perfluoralkyl ether carboxylic acids, HFPO-TA caused a reduction of IL-2 promotor activity at 33 µM (Figure 4).

**FIGURE 6:**
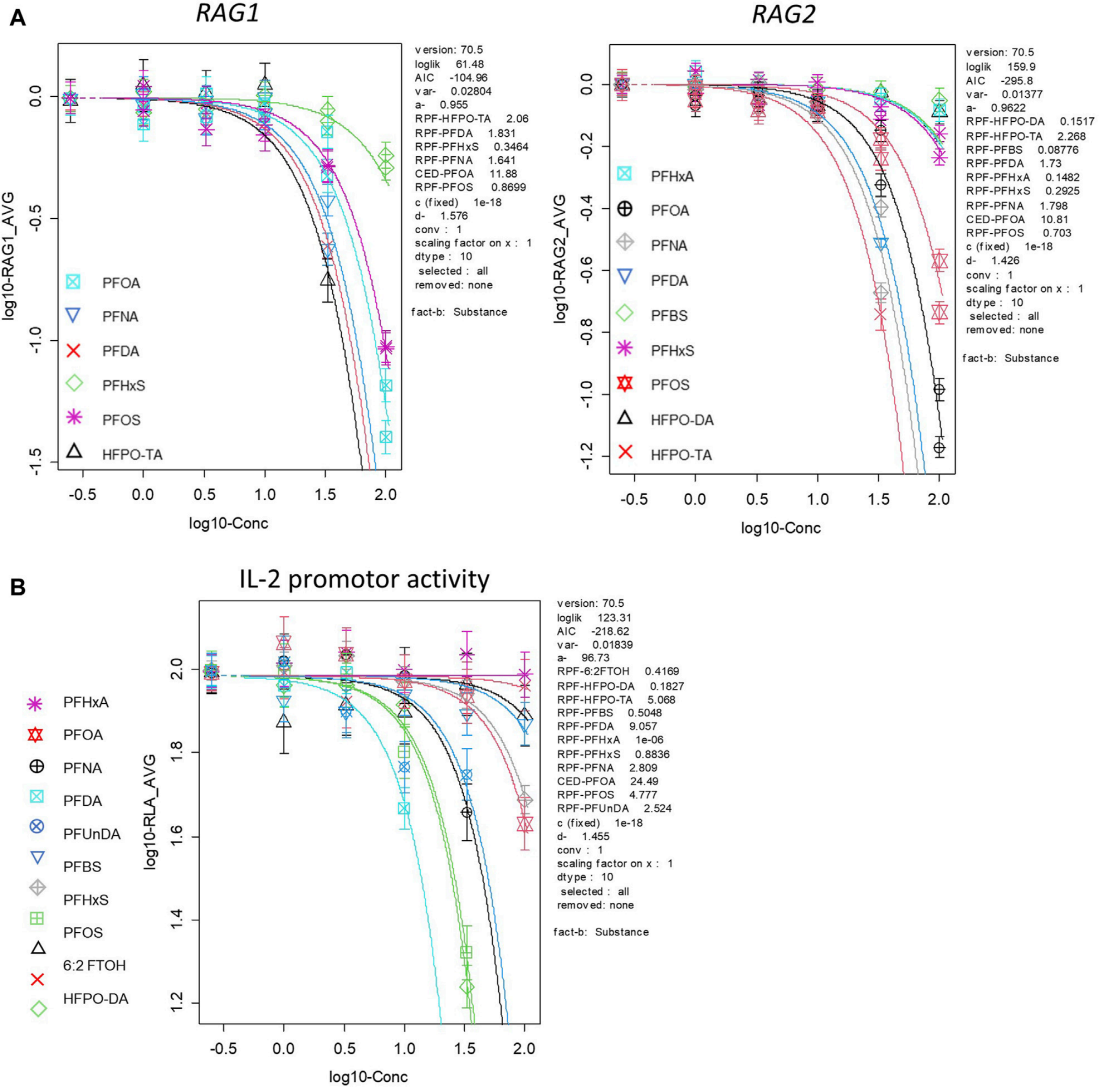
Results of BMD modelling using concentration-effect data regarding **(A)** RAG1 and RAG2 gene expression in Namalwa cells, and **(B)** IL-2 luciferase activity in Jurkat cells. A CES of −0.1 was used, which corresponds to a BMR of 10% (BMR_10_). The observed changes in *RAG1/2* expression and IL-2 reporter activity in response to the PFASs appeared to be best described by the exponential model $y=a*c^{\left.{1-\exp\;(-\left(x/b\right)}^{d}\right)}$ , showing the lowest Akaike information criterion (AIC). The used parameters were a, b, c, and d describing the response at dose 0 (background value), the potency of the PFAS, maximum fold change in response compared with background response (upper or lower plateau), and steepness of the curve (on a log-dose scale), respectively. CES: critical effect size (same as BMR), CED: critical effect dose (same as BMC), CEDL: lower bound of the CED (same as BMCL), CEDU: upper bound of the CED (same as BMCU). Data points represent the mean of triplicates for each PFAS.

To get insight in the relative potencies of PFASs reducing IL-2 promotor activity, concentration-response modeling was performed on the results using PROAST software. Also here, a BMC corresponding to a 10% reduction in relative luciferase activity compared to background level was determined using parallel curve fitting. Figure 6B illustrates the curves fitted by PROAST for the PFASs that yielded relative luciferase activity data adequate for BMD response-modeling. Corresponding BMC_10_ values and RPFs are presented in Table 2. PFDA was the most potent, being 9.1-fold more potent than PFOA in reducing IL-2 promotor activity, followed by HFPO-TA and PFOS, being 5.1-fold and 4.8-fold more potent than PFOA, respectively. PFNA and PFUnDA were also more potent than PFOA (2.8- and 2.5-fold, respectively). The potency of PFHxS was slightly lower than that of PFOA, being 1.1-fold lower*.* HFPO-DA was the least potent of the PFASs able to reduce IL-2 promotor activity, having an RPF of 0.18 (Table 2). Of the tested PFASs, PFBA, PFHxA, 3:1 FTOH, 3:1 FTUOH and 4:2 FTOH did not reduce IL-2 promotor activity.

## Discussion

In the current study, we examined the effects of a diverse group of 15 PFASs on the human B-cell line Namalwa and the human Jurkat IL-2 reporter T-cell line. The aim was to provide mechanistic understanding into PFAS-induced reduced antibody responses, and to obtain insight in possible differences in potencies between the substances. In the Namalwa cells, RPFs could be obtained for 6 PFASs (including the index chemical PFOA) based on their effect on *RAG1* gene expression, and for 9 PFASs on *RAG2* gene expression. In Jurkat T-cells, RPFs could be determined for 10 PFASs based on their inhibitory effect on IL-2 promotor activity. The substances for which RPFs could be derived for all readouts were PFOA, PFNA, PFDA, PFHxS, PFOS, and HFPO-TA. The most potent substances were HFPO-TA for the reduction of RAG1 and RAG2 gene expression in Namalwa cells, with respective RPFs of 2.1 and 2.3 (BMC_10_ of 5.8 μM and 4.8 μM, respectively), whereas in Jurkat cells PFDA was the most potent, being around 9-fold more potent than PFOA (BMC_10_ of 2.7 μM).

To our knowledge, this is the first study investigating a large panel of PFASs, consisting of short- and long-chain perfluorinated carboxylic and sulfonic acids, fluorotelomer alcohols, and perfluoralkyl ether carboxylic acids on immune cells *in vitro*. The perfluorinated carboxylic acids, specifically those with shorter alkyl chain lengths (C4-C6) showed limited to no impact on *RAG1* and *RAG2* expression in Namalwa cells or on IL-2 promotor activity in the Jurkat cells, whereas the perfluorinated carboxylic acids with longer alkyl chain lengths caused effects. Strikingly, the perfluorinated carboxylic acid with the longest chain length tested in this study, PFUnDA (C11) lowered IL-2 promotor activity in Jurkat cells (RPF of 2.5, BMC_10_ of 9.7 μM), but had no effect on *RAG1* and *RAG2* expression in Namalwa cells at the same concentrations tested. Also, this finding could suggest that the effects on IL-2 promoter activity underly a different mode of action than the effects on *RAG1* and *RAG2* gene expression, as also some substances were more potent in decreasing *RAG1* and *RAG2* expression than IL-2 promoter activity, whereas some were similarly potent, and others were more potent in decreasing IL-2 promoter activity than affecting *RAG1* and *RAG2* expression. Alternatively, this could also be driven by differences in distribution of the substances in the different assays systems, and differences in cellular uptake, depending on the cell type. For example, longer chain PFASs bind to serum albumin to a greater extent than shorter chain PFASs (Jackson et al., 2021), which is present in the cell culture medium of experiments with Namalwa cells, but not in experiments with Jurkat cells. This would lead to a lower concentration available for cellular uptake in the Namalwa cells, and could explain the differences observed.

The effect of PFASs on *in vitro* T-cell activation has been assessed in prior studies, yielding conflicting results. In the current study, the induction of IL-2 promotor activity in Jurkat T-cell using anti-CD3/CD28 Dynabeads was reduced by both PFOA and PFOS, with PFOS being 4.8-fold more potent than PFOA. Midgett et al. (2015) also found a significant reduction in IL-2 release by PFOS in Jurkat T-cells, while PFOA had no effect (concentrations of PFOA tested were lower than in the present study and cell culture medium contained 10% FBS). Additionally, this effect was observed in cells stimulated with phytohemagglutinin and phorbol myristate acetate, as well as in anti-CD3 stimulated cells, but not in anti-CD3/CD28 stimulated cells. In a study by Maddalon et al. (Maddalon et al., 2023), PBMCs were stimulated also using anti-CD3/CD28 antibodies and exposed to mixtures containing either PFASs with short alkyl chain lengths (C4-C6), PFASs with long alkyl chain lengths (C8-C9), or a combination of both. Whilst the PFAS mixtures containing only short or long chain alkyl lengths had minor effects on T-cell activation, the mixture containing PFASs of both short and long alkyl chain lengths affected the activation of multiple T-cell populations. Therefore, that study indicates that PFASs may have additive effects, underscoring the need to explore the immunotoxicity of combinations of PFASs as well.

It has been hypothesized that at least part of the immunosuppressive effects of PFASs are mediated by PPARs (peroxisome proliferator activated receptors) (EFSA CONTAM Panel and Scientific Opinion, 2020; Ehrlich et al., 2023). Besides their well-known role in energy homeostasis, PPARs also have immunomodulatory effects, such as a negative regulation of inflammatory responses and decreased generation of inflammatory cytokines (Calder, 2006; Behr et al., 2020; Christofides et al., 2021). Due to their molecular structure, PFASs are agonistic ligands of PPARs [reviewed in (Ehrlich et al., 2023)] of which in particular PPARα and PPARβ/δ are expressed in various types of immune cells, including B- and T-cells (Uhlén et al., 1979; Daynes and Jones, 2002; Bateman et al., 2023). The PFAS-induced decreased expression on *RAG1* and *RAG2* in Namalwa cells and decreased activity of IL-2 reporter activity in Jurkat cells described here are potentially also mediated by PPARs. Upon activation, PPARs are reported to negatively interfere with nuclear factor κB (NF-κB) and (activator protein-1) AP-1 signaling pathways (Chinettl et al., 2000; Harmon et al., 2011; Goujon et al., 2022) and in addition, activated PPAR-γ is reported to physically associate with nuclear factor of activated T-cells (NFAT). Upon activation of the T-cell receptor (TCR), NF-kB, AP-1, and NFAT are stimulated and bind to the canonical response element in the promoter of IL-2, leading to transcriptional regulation of IL-2 gene expression. Interference with these transcriptional regulators can potentially reduce IL-2 promoter activity. It has been demonstrated in mice that PPARγ agonists can reduce levels of IL-2 and other cytokines (Chen et al., 2014). To what extent these factors are involved in the effects observed in the current study remain to be explored further.

RAG1 and RAG2 are target genes of the transcription factor FOXO1, and inactivation of FOXO1, for example, through induction of the PI3K-Akt signaling pathway, is reported to result in decreased expression of the RAG genes (Lazorchak et al., 2010; Lazorchak and Su, 2011; Benhamou et al., 2018; Peña-Pérez et al., 2022). As the results in this study show, inhibition of FOXO1 by AS1842856 indeed also reduced expression of RAG1 and RAG2 in Namalwa cells, which supports that FOXO1 inhibition leads to reduced RAG1 and RAG2 expression. Interestingly, PPARα is reported to be able to antagonize FOXO1 through physical interaction (Qu et al., 2007). Also PPARγ and FOXO1 are reported to interact and inhibit each other in a reciprocal manner (Dowell et al., 2003; Fan et al., 2009). This antagonism of FOXO1 could, at least in part, lead to a reduction in RAG1 and RAG2 expression. Hence, activation of the various PPARs by PFASs and the resulting inhibition of FOXO1 could potentially explain the observed downregulation of *RAG1* and *RAG2* by PFASs in the present study. The concentrations active in reducing *RAG1* and *RAG2* expression, and IL-2 promoter activity are in the same range as concentrations reported to activate PPARs in *in vitro* assays (Behr et al., 2020; Evans et al., 2022), corroborating that for these effects in Namalwa and Jurkat cells, PPAR activation is a plausible explanation. As described before, PPAR is suggested to mediate at least part of the effects observed *in vivo*, though it remains to be established whether this is also the case for the reduced vaccination response.

Another point to be discussed is that the nominal concentrations active in *in vitro* assays are generally higher than for example concentrations of PFASs reported to be found *in vivo* (Heffernan et al., 2018; Ledda et al., 2018; Duffek et al., 2020; Kotlarz et al., 2020; Richterová et al., 2023). For this comparison, the toxicokinetics of PFASs *in vivo*, but also their distribution in *in vitro* systems are important considerations. For *in vitro* assays it is known that cellular uptake of PFASs can be very low, and therefore, relatively high concentrations may be needed for their activity on intracellular targets. It is worth noting that, depending on the type of PFAS and the concentration applied, the uptake (assumed from association with cellular proteins) over 24 h can be as low as below 1% (Bil et al., 2021). In humans, certain PFASs can have long elimination half-lives, and there is continuous exposure due to the widespread presence of various PFASs in the environment and through the diet, resulting in chronic continuous exposure. A better understanding of the *in vivo* kinetics of PFASs in humans can help in the translation and application of *in vitro* data.

In their risk assessment on PFASs in 2020, EFSA derived a TWI for the sum of 4 PFASs (PFOA, PFNA, PFHxS and PFOS). This was based on an inverse association between PFAS levels and antibody titers in the blood of infants, where primarily PFOA and PFOS were detected, and to a minor extent PFNA and PFHxS (Abraham et al., 2020). Similar was reported in a cohort of 5-year-old children from the Faroese Islands (Grandjean et al., 2017). Budtz-Jorgenson et al. (Budtz-Jørgensen and Grandjean, 2023) also reported that in the studies based on the Faroese Island cohorts, PFOS contributed less to the response than PFOA, even though the PFOS levels in those studies were much higher than those for PFOA. This suggests that the relative potency of PFOS for this specific effect on vaccination response is lower than that of PFOA. Peden-Adams *et al.* (Peden-Adams et al., 2008) conducted a study in mice to explore how PFOS impacts the production of antibodies against sheep red blood cells, simulating a T-cell dependent response. It was found that PFOS reduced the antibody response at levels similar to those in the studies with infants and children (Abraham et al., 2020; Bil et al., 2022). Also in other animal studies PFOS, but also PFOA, were shown to have immunotoxic effects, though at higher doses than estimated to be relevant for humans (EFSA CONTAM Panel and Scientific Opinion, 2020). Unfortunately, there are no animal studies comparing effects of different PFASs on the vaccination response, unlike several studies that show effects on the weight of liver and other organs, and on thyroid hormone levels (EFSA CONTAM Panel and Scientific Opinion, 2020; ATSDR, 2021). At this stage the exact mode of action behind the effects of PFASs on TDAR is unclear. In order to allow risk assessment on other than the four aforementioned PFASs, Bil *et al.* proposed RPFs based on liver effects in rodents both for external exposure (23 substances) (Bil et al., 2021) and for blood concentrations (9 substances) (Bil et al., 2022), as well as for lymphoid organ weight and globulin concentration relative to the substances’ blood concentrations (6–9 substances, depending on the endpoint used) (Bil et al., 2023). The RPFs of the effects of PFASs on liver and lymphoid organ weights, based on the internal concentrations were in a similar order, with the marked exception of HFPO-DA that was highly potent for liver effects but did not affect lymphoid organ weight. These differences between RPFs derived from external doses and blood concentrations indicate, among potential differences in the modes of action underlying the effects, also potential differences in the kinetics between the substances. While the gross effects on organ weights at high doses of PFASs are not necessarily directly related to the reduced vaccination responses at lower chronic exposure, they may originate from the same initiating events. Under the (hypothetical) assumption of comparable kinetics at low and high doses, it is of interest to compare the RPFs based on lymphoid organ weights to the *in vitro* RPFs on immune-relevant effects from this study. The RPFs derived on lymphoid organ weights based on blood concentrations were, overall, in decreasing order of potency PFDA > PFNA > PFHxA > PFOS > PFBS > PFOA > PFHxS (ranging from 12 to 0.3). Also in the present study, in particular also PFDA and PFNA were the most potent substances. Moreover, in our study, PFOS reduced IL-2 promoter activity with a higher potency than PFOA, but was slightly less potent in affecting *RAG* gene expression than PFOA. In comparison to the RPFs related to lymphoid organ weights, PFOS was more potently affecting thymus and spleen weight compared to PFOA but was less potently affecting globulin concentrations (Bil et al., 2023). Of the short-chain perfluorinated carboxylic acids, PFHxA and PFBS were both less potent than PFOA in affecting *RAG* expression and IL-2 promotor activity. Interestingly, these substances were affecting thymus weight more potently than PFOA, but spleen weight and globulin concentration less potently than PFOA. Besides the aforementioned absence of an effect of HFPO-DA on lymphoid organ weight, the substance reduced globulin concentrations as potently as PFOA. HFPO-DA reduced *RAG2* gene expression and IL-2 promoter activity only at higher concentrations than PFOA. HFPO-TA, which was not included in the RPFs derived from *in vivo* data, was the most potent substance on *RAG* gene expression, and also very potent in reduced IL-2 reporter activity. The comparison with the 'internal' *in vivo* RPFs show that there is a certain overlap with the *in vitro* RPFs from this study, suggesting that some of the effects observed *in vivo* are possibly underlying the same mechanisms of action as the effects observed *in vitro*.

Overall, the activity and potency of PFASs appears to increase with increasing number of carbon atoms, and at least based on our results on the perfluorinated carboxylic acids until 10 carbon atoms, after which there is a decline in potency with increasing number of carbon atoms. A similar observation was made in which the potency of the effects of PFASs on cell viability and ROS generation in HepG2 cells was increasing with the number of carbon atoms for the PFASs tested (up to 10 carbon atoms) (Amstutz et al., 2022).

Altogether, the current study provides potential mechanistic insights into the reported PFAS-induced decreased antibody response, including a decrease in *RAG1* and *RAG2* gene expression in the human B-cell line Namalwa and a reduction in IL-2 promotor activity in Jurkat T-cells. The presented *in vitro* models may represent useful tools for assessing the immunotoxic potential of PFASs that are relevant for humans, derivation of RPFs and prioritization of substances for further risk assessment.

## Data Availability

The original contributions presented in the study are included in the article/Supplementary Material, further inquiries can be directed to the corresponding author.
