# Supplementary material for: Determination of in vitro immunotoxic potencies of a series of perfluoralkylsubstances (PFASs) in human Namalwa B lymphocyte and human Jurkat T lymphocyte cells
Source: Front Toxicol. 2024 Mar 14;6:1347965. doi: 10.3389/ftox.2024.1347965 (PMC10976438; doi:10.3389/ftox.2024.1347965)
Supplement: Supplementary file 2 [file DataSheet1.docx]

**Supplementary data for:**

**Determination of in vitro immunotoxic potencies of a series of perfluoralkylsubstances (PFASs) in human Namalwa B lymphocyte and human Jurkat T lymphocyte cells**

Aafke WF Janssen^1 *^, Wendy Jansen Holleboom^1^, Deborah Rijkers^1^, Jochem Louisse^1,2^, Sjoerdtje A Hoekstra^1^, Sanne Schild^1^, Misha F Vrolijk^2^, Ron LAP Hoogenboom^1^, Karsten Beekmann^1^

^1^Wageningen Food Safety Research (WFSR), Wageningen University and Research, Akkermaalsbos 2, 6708 WB Wageningen, the Netherlands.

^2^European Food Safety Authority, Parma, Italy.

^2^Department of Pharmacology and Toxicology, Maastricht University, 6229 ER Maastricht, the Netherlands.

***Corresponding author:**

Aafke W.F. Janssen PhD

Wageningen Food Safety Research

Akkermaalsbos 2

6708 WB Wageningen

The Netherlands

Phone: +31 317 483670

Email: [aafke.janssen@wur.nl](mailto:aafke.janssen@wur.nl)

A

B

**Supplementary Figure 1. Concentration-dependent effects of deoxynivalenol on viability, and RAG1/2 gene expression in Namalwa cells. (A)** Namalwa cells were exposed to 0.33, 1, 3.3 or 10 µM DON for 48 h. Viability was determined using the WST-1 assay and expressed as percentage of solvent control (0.1% Ethanol). **(B)** Relative expression of *RAG1* and *RAG2* after incubation of Namalwa cells with 3.3, 1 and 0.33 µM for 48 h. Gene expression levels of the solvent controls were set at one. ** P<0.001. Data are presented as mean ± SD from triplicate wells.

B

A

**Supplementary Figure 2. Concentration-dependent effects of CD3/CD28 dynabeads on viability, and IL-2 luciferase activity in Jurkat T-cells. (A)** Jurkat IL-2 reporter T-cells were exposed to CD3/CD28 Dynabeads for 24 h at the following beads-to-cells ratio: 1:1, 2:1, 4:1 and 8:1. Viability was determined using the WST-1 assay and expressed as percentage of Jurkat IL-2 reporter T-cells exposed to solvent control (0:1). **(B)** Luciferase activity of Jurkat IL-2 reporter T-cells incubated with 1:1, 2:1, 4:1 and 8:1 beads-to-cells ratio CD3/CD28 dynabeads for 24 h compared to luciferase activity of Jurkat IL-2 reporter T-cell not incubated with CD3/CD28 Dynabeads. ** P<0.001. Data are presented as mean ± SD from triplicate wells for the cell viability assays (n=3) and from sextuplicate wells for the luciferase activity studies (n=6).

B

A

**Supplementary figure 3. Concentration-dependent effects of FK506 on viability, and IL-2 luciferase activity in Jurkat T-cells. (A)** Jurkat cells were exposed to 3.3, 10, 33 or 100 nM FK506 for 24 h. Viability was determined using the WST-1 assay and expressed as percentage of solvent control (0.1% DMSO). **(B)** Luciferase activity of Jurkat IL-2 reporter T-cells exposed to 0.1, 1 or 10 nM FK506 for 1 hour after which CD3/CD28 Dynabeads were added (8:1 beads-to-cells ratio) for 24 h. IL-2 luciferase activity of the solvent controls were set at 100%. ** P<0.001. Data are presented as mean ± SD from triplicate wells for the cell viability assays (n=3) and from sextuplicate wells for the luciferase activity studies (n=6).

*Supplementary Table 1. Suppliers, purities, catalog numbers, CAS numbers and maximum concentrations of chemicals tested in the present study.*

| **Chemical** | **Full name** | **Supplier** | **Purity** | **Catalog number** | **CAS number** | **Highest tested concentration (µM)** |
| --- | --- | --- | --- | --- | --- | --- |
| PFBA | Perfluorobutanoic acid | Sigma-Aldrich | ≥98% | 68808-25MG | 375-22-4 | 100 |
| PFHxA | Perfluorohexanoic acid | Synquest laboratories | 97% | 2121-3-39 | 307-24-4 | 100 |
| PFOA | Perfluorooctanoic acid | Sigma-Aldrich | 98.8% | 171468-5G | 335-67-1 | 100 |
| PFNA | Perfluorononanoic acid | Sigma-Aldrich | 99.7% | 91977-50MG | 375-95-1 | 100 |
| PFDA | Perfluorodecanoic acid | Sigma-Aldrich | 98% | 177741-5G | 335-76-2 | 100 |
| PFUnDA | Perfluoroundecanoic acid | Sigma-Aldrich | 100% | 446777-5G | 2058-94-8 | 33 |
| PFBS | Perfluorobutane sulfonate | Sigma-Aldrich | 100% | 562629-5G | 375-73-5 | 100 |
| PFHxS | Perfluorohexane sulfonate | Synquest laboratories | 95% | 6164-3-2T | 355-46-4 | 100 |
| PFOS | Perfluorooctane sulfonate | Synquest laboratories | 100% | 6164-3-08 | 1763-23-1 | 100 |
| 3:1 FTOH | 2,2,3,3,4,4,4-Heptafluoro-1-butanol | Synquest laboratories | 99% | 2101-3-12 | 375-01-9 | 100 |
| 3:1 FTUOH | 2,2,3,4,4,4-Hexafluoro-1-butanol | Sigma-Aldrich | 95% | BL3H97A4923A-25G | 382-31-0 | 100 |
| 4:2 FTOH | 3,3,4,4,5,5,6,6,6-Nonafluoro-1-hexanol | Synquest laboratories | 99% | 2101-3-95 | 2043-47-2 | 100 |
| 6:2 FTOH | 3,3,4,4,5,5,6,6,7,7,8,8,8-Tridecafluoro-1-octanol | Synquest laboratories | 99% | 2101-3-20 | 647-42-7 | 100 |
| HFPO-DA (GenX) | Hexafluoropropylene oxide dimer acid | Synquest laboratories | 98% | 2121-3-13 | 13252-13-6 | 100 |
| HFPO-TA | Hexafluoropropylene oxide trimer acid | abcr GmbH | 97% | 164194-5G | 13252-14-7 | 100 |
| AS1842856 | - | Sigma-Aldrich | ≥98% | 344355-10MG | - | 1 x 10^-5^ |
| DON | Deoxynivalenol | Sigma-Aldrich | 100% | D0156-1MG | 51481-10-8 | 10 |
| FK506 | - | Sigma-Aldrich | ≥99% | F4679-5MG | 109581-93-3 | 0.01 |
